# Shrimp Allergy and Its Impact on a Career in the Armed Forces

**DOI:** 10.7759/cureus.92292

**Published:** 2025-09-14

**Authors:** Anju Sivadasan, Leyla Ozyigit, Nasreen Khan, Shuaib Nasser

**Affiliations:** 1 Allergy and Immunology, Leicester Royal Infirmary, University Hospitals of Leicester NHS Trust, Leicester, GBR; 2 Allergy, Glenfield Hospital, University Hospitals of Leicester NHS Trust, Leicester, GBR; 3 Allergy, Addenbrooke's Hospital, Cambridge University Hospitals NHS Foundation Trust, Cambridge, GBR

**Keywords:** anaphylaxis, food allergy, military occupational health guidelines, oral food challenge, shrimp allergy, skin prick testing, tropomyosin

## Abstract

A diagnosis of food allergy can significantly affect the career prospects of military aspirants, as severe allergic reactions and the need to carry an adrenaline auto-injector are among the medical conditions that disqualify individuals from military service. Uncertainty regarding fitness for military recruitment may include candidates who were given a label of food allergy in early life with no recent reactions, those with mild oral symptoms when exposed to certain foods but recorded as allergic, others who have been prescribed adrenaline auto-injectors with an unclear indication, and those with childhood food allergies that may have resolved. Shellfish allergy is a prevalent and potentially fatal food allergy that typically persists throughout life. This case report describes a man in his early 20s with suspected prawn allergy, which became a concern during his job application to the Royal Navy. Despite positive skin prick tests and specific IgE levels indicating sensitization, the patient successfully passed an oral food challenge without reaction. This permitted the removal of the shellfish allergy diagnosis, allowing him to proceed with his military job application. This case highlights the importance of comprehensive allergy testing and food challenges in diagnosing food allergies, especially in cases with significant implications for career opportunities.

## Introduction

Shellfish are one of the most common food groups responsible for food allergies, with an estimated prevalence of up to 2.8% in the general population [1]. The clinical manifestations of shellfish allergy can range from mild skin symptoms to severe anaphylaxis. Data suggest that shellfish allergy is also one of the common causes of fatal food allergy [2]. Additionally, shellfish allergy appears to persist throughout life, with a low rate of resolution compared to other food allergies [3]. Although diagnosis usually involves taking a patient's medical history, carrying out skin prick tests, and testing for specific IgE, it can be difficult to distinguish true sensitization from cross-reactivity with other invertebrates or inhalant allergens. Moreover, new findings in molecular allergology and component-resolved diagnostics are changing our view of shrimp allergy phenotypes, prognosis, and risk stratification [4].

Crustaceans, including shrimp, crab, and lobster, are classified as arthropods together with mites, spiders, and insects. This likely provides an explanation for their observed molecular and allergenic clinical cross-reactivity [4,5]. Tropomyosin is a heat-stable pan-allergen in invertebrates [6]. Several studies have shown that tropomyosin is responsible for IgE cross-reactivity among crustaceans, mollusks, cockroaches, *Anisakis simplex* (a common fish parasite), and *Dermatophagoides pteronyssinus* (house dust mite). Sensitization can occur by ingestion (seafood), inhalation (mites, cockroaches, occupational exposure during shellfish processing), or skin contact with food sources [4].

Severe allergic reactions and a requirement to carry adrenaline auto-injectors (AAIs) are included among the medical conditions that preclude joining the military as a career [7]. In the UK, if a military applicant passes initial scrutiny, an army-approved doctor will review their health records to see if they have any conditions that would be a barrier to recruitment [8]. If there is any mention of food allergy in the medical record, this will be flagged, and the person will need to be assessed by an approved allergist.

We present a case report of a patient in this situation with concern around shrimp allergy and a review of current knowledge on this topic.

## Case presentation

A 22-year-old man hoping to join the Royal Navy was referred to us due to an entry in his medical records indicating a possible prawn allergy. His mother recalled that at age 10 years, he developed a strange sensation in his lips and mouth after eating a prawn curry. No treatment was needed for this episode. When he joined a military boarding school in 2017, this information was conveyed to the school nurse, which resulted in him being recorded as shellfish-allergic. He had no history of symptoms such as throat tightness, difficulty breathing, or collapse suggestive of a systemic allergic reaction or anaphylaxis.

He had mild perennial allergic rhinitis as a child. There was no history of asthma or atopic dermatitis. He ate a varied diet that included fish, while avoiding prawns and other crustaceans. He was otherwise fit and well, with a normal physical examination, and was not on any regular medications.

His skin prick tests (SPT) were performed with standard solutions (ALK-Abello, Horsholm, Denmark) and interpreted according to European standards during his first assessment [9]. The diameters of the wheals were as follows: D. pteronyssinus (house dust mite) 11 mm; mixed grasses 6 mm; birch tree 0 mm; shrimp 10 mm; mussel 4 mm. The positive (1% histamine hydrochloride) control was 5 mm, and the negative control (0.9% normal saline solution) was 0 mm. Serum-specific IgE levels were quantified using ImmunoCAP™ (Thermo Fisher Scientific, Waltham, Massachusetts), with results summarized against a total IgE level of 259 kU/L (Table 1).

**Table 1:** The list of results for specific IgE levels tested using the ImmunoCap method (normal value: <0.35 kUA/L) IgE: immunoglobulin E, kUA/L: kilounits of allergen-specific IgE per liter.

Allergen	Specific IgE (kUA/L)
Shrimp	9.59
Squid	0.44
Blue mussel	1.74
Anisakis simplex	1.79
*rPen a 1* tropomyosin shrimp	6.05
*rDer p 10* tropomyosin house dust mite	4.86

Prick-prick tests were positive for *Penaeus monodon* (Black Tiger Prawn; 10 mm) and *Pandalus borealis* (Caridean Shrimp; 9 mm).

The case was discussed in detail during our multidisciplinary team meeting, with particular emphasis on the ethical considerations of duty of care versus patient safety when considering an oral food challenge (OFC) to prawn. There was agreement that detailed informed consent was required before proceeding to an oral food challenge. After shared decision-making with the patient, he underwent an open graded food challenge to cooked prawn. A mixture of Black Tiger Prawn and Caridean Shrimp was used, to include both warm-water and cold-water varieties of prawns. Equal proportions of the two types of prawns were used to make a total dose of 10 grams. The test was performed under close observation and monitoring in the day-case unit of the Adult Allergy Service. A four-dose oral food challenge protocol (with 20-minute intervals) was used, according to guidelines [10]. The patient was observed for 1 hour after completing the total dose. He tolerated prawn without symptoms or signs of an allergic reaction.

Our advice was that the label of shellfish allergy should be removed from the patient’s records, and no further investigation was required from the Adult Allergy Service. This enabled the patient to proceed with his job application to the Royal Navy.

## Discussion

This case highlights the complexities of diagnosing shellfish allergy and the potential occupational impact of an unverified allergy label. While the oral food challenge remains the reference diagnostic procedure for confirming or excluding food allergy, it must be preceded by a thorough history, in vitro and in vivo testing, and shared decision-making regarding whether to perform an oral food challenge [11]. In addition to skin prick tests with standard solutions, prick-to-prick tests using different types of raw and cooked shrimp can be performed.

Geographical location, dietary habits, and co-sensitization to house dust mite may have an impact on sensitization patterns in shellfish allergy [12]. According to a recent food allergy guideline, the sensitivity of shrimp-specific IgE is considered to be high (96%) with a cutoff of 1.2 kU/L [13]. Skin and blood test results require careful interpretation with consideration of cross-reactivities, especially between mite and crustacean allergens, as is likely in this case.

In a Singapore-based cohort of shrimp-allergic patients (confirmed by food challenge), shrimp-specific IgE was found to have a sensitivity of only 62% and a specificity of 50% for detecting allergy [14]. In the presence of mite allergy, the specificity and clinical predictive value of shrimp-specific IgE testing are even lower [15]. Component analysis, however, may improve specificity [16]. A study of 35 patients in São Paulo demonstrated that the specificity of measuring IgE to shrimp tropomyosin Pen a 1 (92.8%) was greater than that of shrimp-specific IgE (75%) and skin prick testing (64.2%) [17].

Individuals initially sensitized to house dust mite tropomyosin Der p 10 via inhalation may subsequently develop sensitization to shellfish allergens. It has been hypothesized that when the primary sensitizer is a tropomyosin from an inhaled source, such as house dust mite tropomyosin, symptoms may predominantly manifest as oral symptoms [18].

As demonstrated in our patient, sensitization to shrimp and tropomyosin without a clear history of allergy illustrates the clinical dilemma posed by cross-reactivity. An oral food challenge is required to comprehensively rule out allergy. Successfully passing the oral shrimp challenge allowed our patient to confidently pursue his military vocation.

The United States Department of Defense Instruction (DoDI) guidelines state that a "history of systemic allergic reaction to food or food additives" is disqualifying [19]. In 2018, the Military Allergy and Immunology Assembly (MAIA) of the American Academy of Allergy, Asthma & Immunology (AAAAI) published guidance on food allergy evaluation and management for civilian and military practice [7]. According to these guidelines, US military applicants with a history of food allergy require allergist assessment prior to application submission. If the allergist recommends adrenaline auto-injector carriage, or if either the allergist or applicant is unable or unwilling to perform a food challenge based on reaction risk, the service-specific allergy consultant is unlikely to recommend a waiver. However, if the applicant is able to pass an oral food challenge without reaction, then the food allergy is considered resolved, and a waiver is recommended despite a previous reaction history, including anaphylaxis. The United States Department of the Air Force (DAF) issued a policy update in December 2024, which states that applicants with a history of food allergies, provided there has been no anaphylaxis or serious systemic reaction, will now qualify for a waiver [20].

In a study published by Thong et al. in 2018, the authors found that allergy to shellfish/crustaceans was the most common food allergy among military pre-enlistees in Singapore, and oral allergy syndrome (OAS) to shellfish/crustaceans was more common than anaphylaxis [21]. OAS typically presents with mild, localized symptoms such as itching or tingling of the lips, mouth, and throat without systemic involvement. The authors concluded that for most shellfish/crustacean-allergic pre-enlistees who have mild oropharyngeal symptoms, the avoidance of seafood ingredients in field rations should be sufficient to allow them to be field-deployable, as the risk of anaphylaxis in this group is likely to be low. However, for service personnel with shellfish anaphylaxis, the risk of accidental ingestion during field work and timely access to emergency medical attention limited deployability. The authors advised performing OFCs to evaluate anaphylaxis risk and determine deployability in individuals with oral allergy syndrome.

Areas of uncertainty with regard to fitness for military recruitment include candidates who were given a label of food allergy in early life but have not had any recent reactions and may not actually have an allergy, individuals who only experience mild oral symptoms on exposure to food (oral allergy syndrome) but whose medical records state that they are allergic, and others who have been prescribed an AAI as a precaution but the indication is unclear and they have never needed to use it [8]. Other patients may have had mild food allergy in childhood but may have grown out of it. These categories of patients may need a formal oral food challenge after initial clinical and laboratory assessment to show that the diagnosis of allergy is no longer valid or is so mild that it does not present a problem for recruitment into the armed forces.

This case highlights the importance of accurate diagnosis and management of food allergies in occupational settings, particularly in professions with stringent health requirements such as the military. Ensuring that individuals are not inappropriately excluded due to misdiagnosed allergies can facilitate optimal workforce participation while maintaining safety standards.

## Conclusions

Military aspirants with a medical history of allergy require formal allergy assessment via approved allergy services. Severe allergic reactions necessitating the carriage of adrenaline auto-injectors can disqualify potential candidates. A formal oral food challenge after clinical evaluation and risk assessment may be required for candidates with an unclear or unsubstantiated history of food allergy. Reliance solely on initial tests can lead to misdiagnosis, resulting in unnecessary lifestyle or career restrictions. The presence of cross-reactivity between crustaceans and other arthropods, such as mites and some insects, due to shared allergens like tropomyosin, can complicate the interpretation of allergy tests. Understanding this cross-reactivity is crucial for accurate diagnosis and management.
